# PREVALENCE OF GENU VALGUM IN PUBLIC ELEMENTARY SCHOOLS IN THE CITY OF SANTOS (SP), BRAZIL

**DOI:** 10.1590/1984-0462/;2017;35;4;00002

**Published:** 2017-09-21

**Authors:** Maria Célia Cunha Ciaccia, Camila Nazareth Pinto, Fernanda da Costa Golfieri, Tales Ferreira Machado, Lívia Lopes Lozano, João Marcel Sanseverino Silva, Vera Esteves Vagnozzi Rullo

**Affiliations:** a Centro Universitário Lusíada de Santos, SP, Brasil.

**Keywords:** Genu valgum, Prevalence, Obesity, School health, Genuvalgo, Prevalência, Obesidade, Saúde escolar

## Abstract

**Objective:**

To evaluate the prevalence of *genu valgum* and associated factors in elementary school students.

**Methods:**

Cross-sectional study, carried out in 2015, with 1,050 children and adolescents enrolled in an elementary school in Santos, Southeast Brazil. Misalignment of the knee was assessed by intermalleolar distance, considering ≥8 cm or <8 cm. Inter-examiners reliability was measured by Kappa coefficient, resulting in 0.94. Nutritional status was evaluated according to the World Health Organization 2006 references. Logistic regression model was applied to analyze variables associated with *genu valgum.*

**Results:**

Among schoolchildren, 7.1% had *genu valgum.* The frequency was higher among overweight or obese schoolchildren. On average, students with *genu valgum* are older than those without it. There was no association with gender. Upon logistic regression model, only nutritional status was significantly associated with this condition. The chance of occurrence of valgus knee in overweight and obese schoolchildren was, respectively, 6.0 and 75.7 times greater than among thin or eutrophic subjects.

**Conclusions:**

The prevalence of *genu valgum* in elementary school children and adolescents was 7.1%, being higher among overweight/obese students and presenting no association with gender or age.

## RESUMO

**Objetivo:**

Avaliar a prevalência de genuvalgo e fatores associados em alunos do ensino fundamental.

**Métodos:**

Estudo transversal, realizado em 2015, com 1.050 crianças e adolescentes matriculados no ensino fundamental de Santos. Foi avaliado o desalinhamento do joelho, medindo-se a distância intermaleolar, considerada se ≥≥8 cm ou <8 cm. O coeficiente *Kappa* de 0,94 foi obtido para aferir a confiabilidade dos exames entre os avaliadores. O estado nutricional foi verificado segundo a Organização Mundial de Saúde, 2006. Aplicou-se modelo de regressão logística para avaliar fatores associados à presença de genuvalgo.

**Resultados:**

Dos escolares analisados, 7,1% apresentaram genuvalgo. Aqueles com sobrepeso ou obesos apresentaram porcentagens maiores de genuvalgo. Em média, os estudantes com genuvalgo são mais velhos do que aqueles sem genuvalgo. Não foi verificada associação com o sexo. No modelo de regressão logística, apenas a avaliação nutricional permaneceu significantemente associada à presença de genuvalgo. A chance de sua ocorrência em escolares com sobrepeso e obesos é, respectivamente, 6,0 e 75,7 vezes maior que a chance de ocorrência em magros ou eutróficos.

**Conclusões:**

A prevalência de genuvalgo em crianças e adolescentes do ensino fundamental da rede municipal de Santos foi de 7,1%, sendo maior nos alunos com sobrepeso/obesos, não apresentando associação com o sexo ou a idade.

## INTRODUCTION

*Genu valgum* or valgus knee is a deformity resulting from the separation of ankles, when the medial faces of the knees are in contact while the person is in anatomical position, the patella and the hallux turning to the anterior direction.^1^ In children aging 2 to 6 years, valgus knee is normal within certain limits of knee angle, therefore being characterized as physiological.^2^ Most children presenting this condition at these ages have spontaneous correction.^3^ Angle and type of deviation can be either normal or physiological, depending on the age.^4^ Newborns are usually defined as physiological and, around 18 to 24 months, their tibiofemoral angle (TFA) variation aligns to zero degree. At 3 years, the maximum deviation value in children with normal development reaches 12 degrees and decreases until it stabilizes at 5 to 6 degrees by 6 or 7 years of age.^4^ Intermalleolar distance measured with a ruler, being the child in orthostatic position, is one of the clinical alternatives to measure valgus deformity.^3^ ^,^ ^5^ Children under 7 years presenting physiological valgus knee have Intermalleolar distance of up to 8 cm, the greatest distance being observed between 3 and 4 years.^6^

The prevalence of obesity is known to have been increasing over the last decades. According to Calvete,^7^ obesity causes mechanical overload to the locomotor system, postural misalignment with anteriority of mass center, thus leading to feet functional alterations and an increase in mechanical needs to adapt to the new body scheme. The action of body weight on the feet makes the medial longitudinal arch tend to fall, assuming a pronated or valgus foot posture. In order to compensate, the tibia happens to rotate internally and, consequently, there is knee compression, pain and medial compartment wear, as well as an internal rotation of the hip, which contributes to a greater vector in knee valgus and misalignment of the extensor system.^8^

Kapandji^9^ states that lateral deviations of the knee cause arthrosis over time because the imposed loads are no longer equally distributed between the outer and the inner halves of the knee. Such valgus deformities can determine dysfunctions of the lower limbs with relevant consequences in daily activities, including walking, sitting and raising, going up and down stairs.^1^

Knowing the prevalence of valgus knee in school age is of paramount importance so that one can create and implement strategies for prevention and monitoring of future problems. Thus, the purpose of this study was to verify the prevalence of *genu valgum* in elementary school children and adolescents, and to associate it with age, gender, and body mass index (BMI).

## METHOD

This is a cross-sectional study carried out during the 2015 school year, in the municipal network of schools of Santos, with primary education children and adolescents attending 1st to 5th grades. This group was chosen because it included children older than the age group with the greatest intermalleolar distance observed for diagnosis of physiological valgus knee, and the adolescents were below the age group that usually presents increased prevalence of this condition.^10^ Children and adolescents with any known orthopedic pathology or who, for some reason, had already gone through any previous orthopedic treatment were excluded from the sample.

Knee misalignment was measured by intermalleolar distance, using a ruler graduated in centimeters. Students were in orthostatic position with observation in posteroanterior direction, as recommended by Heath and Staheli.^6^ Most studies show a variation in intermalleolar measures according to age, but there is still no consensus on normality patterns.^3^ ^,^ ^6^ ^,^ ^10^ Thus, regardless of age, values ≥ 8 cm or <8 cm were considered for the assessment, as suggested by Heath and Staheli,^6^ who deemed up to 8 cm normal for children aging 2 to 11 years.

Concomitantly, anthropometric measures of weight and height were used in search of associated factors. In order to measure weight, a mechanical Geon scale with graduation of 100g and capacity for 150 kg was used. The child was not supposed to be wearing coats and should be barefoot, that is, in as little clothing as possible. For stature measurement, a wall stadiometer was used. Nutritional status was evaluated by means BMI/age, in Z score, complying with the 2007 Reference Curves of the World Health Organization.^11^

Five medical students were trained to apply the anthropometric measures and the intermalleolar distance in order to collect data, which were always evaluated by two observers - three, in case of disagreement. All measurements were taken at the school itself. In order to evaluate the reliability of intermalleolar distance by all evaluators, Kappa coefficient was applied (0.94) and showed strong agreement between them.

Epi Info 6 (November 1996) was used for sample calculation. Out of 14,732 children enrolled in primary education from 1st to 5th grades at municipal schools in Santos, a sample of 979 children was selected. Sample size was calculated on the basis of 56.6% expected frequency (anchored in the study by Souza *et al*.^12^ carried out in 2013, Ilhabela, São Paulo), with error margin of 3% and confidence interval of 95% (95%CI).

After approval by the Research Ethics Committee of Lusíada University Center and authorization by the Municipal Health and Education Department of Santos, 12 out of 34 public schools assisting students from 1st to 5th grades were drawn. Ten students in ten classes were then randomly selected for the research. After authorization by school directors, children were invited to take part in the study. Informed consent form was signed by caregivers/parents and, once approved, data started being collected, with good acceptance and collaboration of all subjects.

At first, data were descriptively analyzed. Categorical variables were presented as absolute and relative frequencies, and numerical variables as mean and standard deviation. Association between two categorical variables was found after chi-square test was applied. Comparison of means between two groups was made with Student’s t-test for independent samples. Then, in order to evaluate simultaneous effects of possible risk factors (predictor variables) of valgus knee (dependent variable), the logistic regression model was adjusted. Gender, age and BMI were considered as predictors. All variables were initially included in the model; then variables with level of significance less than 5% were one by one excluded, from the highest to the lowest value (backward method). In addition, the adjustment of final model adequacy was assessed by Hosmer and Lemeshow’s test. For all statistical tests, significance level was set at 5%. SPSS Statistics (v.20, IBM SPSS, Chicago, IL) and Stata 12 (Stata Statistical Software: Release 12. College Station, TX: StataCorp LP) were the statistical programs used in the analysis.

## RESULTS

A total of 1,050 adolescents and children were evaluated, 71 more than the calculated sample, mean age being 8.5±1.6 years, ranging from 5 to 13 years. 7.1% (95%CI 5.7-8.9) of children and adolescents actually presented valgus knee. As for gender, 52% of participants were females. Nutritional status evaluation divided the subjects as follows: 1.9% thin, 57.4% eutrophic, 16.9% overweight, and 23.8% obese.

Table 1 shows the distribution of children and adolescents according to age, gender, and nutritional status. *Genu valgum* presented no association with gender, however it was shown to related to nutritional status, meaning that the condition was more common in overweight or obese children and adolescents. Higher percentages of occurrence were found among older students, even though there was no association between age and valgus knee (*p*=0.116).

**Table 1:**
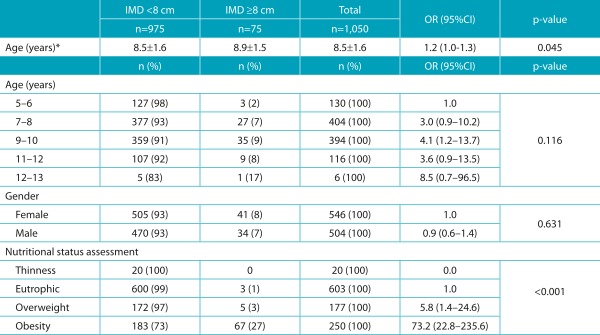
Distribution of children and adolescents according to presence or absence of intermalleolar distance ≥8 cm and to variables such as age, gender and nutritional status.

IMD: intermalleolar distance; OR: Odds Ratio; 95%CI: 95% confidence interval; *expressed as mean?standard deviation.

A logistic regression model was then adjusted, with the presence of *genu valgum* as the dependent variable and, as explanatory variables, sex, age and nutritional assessment. The categories with the highest number of cases were categorized as predictors of categorical predictors. The category “leanness” was added to that of “eutrophic” due to the small number of cases. Table 2 shows two logistic regression models: the initial and the final. The final model presented a good fit of fit according to the Hosmer and Lemeshow test (*p*=1,000). Only nutritional evaluation remained significant in the model. Thus, the chance of occurrence of valgus knee in overweight schoolchildren is 6.0 times higher than among thin or eutrophic children. Likewise, obese patients have a 75.7 times higher chance of developing this condition compared to thin or eutrophic subjects.

**Table 2:**
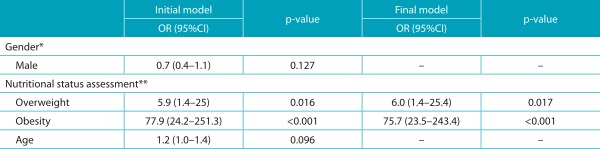
Results of logistic regression models with presence of *genu valgum* as dependent variable (intermalleolar distance ≥8 cm) and gender, age and nutritional status as explanatory variables.

OR: Odds Ratio; 95%CI: 95% confidence interval; *reference: female gender; **reference: thinness/eutrophy.

## DISCUSSION

The prevalence of valgus knee in this study (7.1%) was lower than that found by Souza et al.,^12^ (56.6%), but higher than the results by Aparício et al.^13^ (6.5%).

Bivariate analysis showed no relation with gender, but there was directly proportional association with age, that is, a increase in the frequency of valgus knee as age increased, as also noted by Souza et al.^12^ and Aparício et al.^13^ For Souza et al.,^12^ the youngest children presenting the condition show difficulty in performing physical activities, which increases the the chance of overweight over the years. Therefore, it seems that the primary associated factor is overweight, and not aging, as it was verified in the multivariate analysis.

The World Health Organization considers obesity a global epidemic of increasing prevalence. Nowadays, it’s seen as the most common disease in the world that may lead to diverse comorbidities, even in the pediatric population.^14^ Similarly to our findings, the association between *genu valgum* and obesity was also stated by other studies. Souza et al.^12^ reported that the higher the valgus degree, the more relevant is obesity, and the lower the valgus degree, the lower the adiposity degree. Silva et al.^15^ also link higher values of intermalleolar distance with overweight. Landauer et al.^16^ noted that valgus with femoral deviation is common in obese youngsters. Serra et al.^17^ and Jankowicz-Szymanska and Mikolajczyk^18^ reported prevalence of valgus knee in obese children compared to non-obese children. Jannini et al.^19^ and Brandalize and Leite^20^ have also pointed out that obesity can cause deformities in the lower limbs, including *genu valgum*.

The association between obesity and valgus knee is explained by a sum of factors. Wearing et al. reported that bone tissues remodel according to load exerted on it and, in childhood, they have a greater amount of collagen, therefore being more flexible, more tolerant to plastic deformation, and less resistant to compression. Thus, when overload is increased - as it happens with obese individuals - , those who are in growth phase are more susceptible to deformities.^16^

Bruschini and Nery explain this by indicating that the presence of abdominal protrusion in the obese causes the center of gravity to move anteriorly, leading the vertebral column and the lower limbs to adjust, with pelvic anteversion associated with internal rotation of the hips. All this, along with fat accumulation at the thigh region, causes the malleolus to move away, promoting medial half opening and hyper pressure to the lateral half of the knee. Over time and development, uneven growth occurs between both parts and the deformity installs permanently.

Despite having a representative sample of students from municipal schools in Santos, this is a transversal study, so it does not address other variables that could influence the presence of valgus knee. Other factors such as race, lifestyle, posture across different daily activities of study and leisure should be evaluated, with emphasis to ergonomics, different daily physical activities among children of both genders and different ages, even at puberty, which occurs in distinct age groups between boys and girls, for it all leads them to acquire different body postures. Finally, further cohort studies involving these aspects are strongly recommended so that a cause-effect relationship can be better established.

Knowing the prevalence of valgus knee can raise awareness of the problem and alert to the creation and implementation of strategies that can help avoid major disorders in the future. Association with overweight signals the importance of eating habits education and physical activity in schools, as obesity is currently considered a worldwide outbreak.

Conclusion is that the prevalence of *genu valgum* in elementary school children and adolescents from the municipal network of the city of Santos was 7.1%, being higher among the obese and showing no association with gender or aging.
